# Supplementary material for: Unemployment Trajectories and the Early Risk of Disability Pension among Young People with and without Autism Spectrum Disorder: A Nationwide Study in Sweden
Source: Int J Environ Res Public Health. 2020 Apr 5;17(7):2486. doi: 10.3390/ijerph17072486 (PMC7177271; doi:10.3390/ijerph17072486)
Supplement: Supplementary file 1 [file ijerph-17-02486-s001.pdf]

International Journal of
Environmental Research and Public Health

MDPI

Supplementary Materials: The following are available online at www.mdpi.com/xxx/s1, Table S1: Trajectory solution justification. BIC values of the simpler and more complex models and the log Bayesian factors, Table S2: Mean days of unemployment per year among people with autism spectrum disorder (ASD), their matched references in the total population, and by the trajectory groups. Figure S1: Unemployment trajectory during the diagnosis year (0) and a four-year follow-up among individuals with autism spectrum disorder (ASD, n = 814). AVG = average (solid line), PRED = predicted for the identified trajectory group (dashed line with 95% confidence intervals). Cubic term was statistically significant for the shape of the trajectory.

Table S1. Trajectory solution justification. The Bayesian Information Criterion (BIC) values of the simpler and more complex models and the log Bayesian factors.

	BIC of the complex model	BIC of the simpler model	**log Bayes factor**
Individuals with autism spectrum disorder and their matched references			
2 trajectory group vs. 1 group solution	-45214.3	-51752.2	13075.8
3 trajectory group vs. 2 group solution	-44862.9	-45214.3	702.7

Table S2. Mean days of unemployment per year among people with autism spectrum disorder (ASD), their matched references in the total population, and by trajectory group.

Unemployment days during the year of the follow-up start, and four years after	N	Mean days per year	Lower 95% CI	Upper 95% CI
**All**				
Unemployment days the year of follow-up start	22827	19.19	18.57	19.81
Unemployment days year 1 after follow-up start	22827	17.68	17.06	18.30
Unemployment days year 2 after follow-up start	22827	14.25	13.68	14.81
Unemployment days year 3 after follow-up start	22827	12.80	12.25	13.36
Unemployment days year 4 after follow-up start	22827	10.88	10.37	11.39
**Trajectory group 1**				
Unemployment days the year of follow-up start	1883	0	.	.
Unemployment days year 1 after follow-up start	1883	0	.	.
Unemployment days year 2 after follow-up start	1883	27.57	24.93	30.20
Unemployment days year 3 after follow-up start	1883	57.30	54.04	60.57
Unemployment days year 4 after follow-up start	1883	52.65	49.38	55.93
**Trajectory group 2**				
Unemployment days the year of follow-up start	16247	6.62	6.22	7.03
Unemployment days year 1 after follow-up start	16247	2.99	2.73	3.25
Unemployment days year 2 after follow-up start	16247	2.13	1.90	2.36
Unemployment days year 3 after follow-up start	16247	0	.	.
Unemployment days year 4 after follow-up start	16247	0	.	.
**Trajectory group 3**				
Unemployment days the year of follow-up start	4697	70.34	68.24	72.44
Unemployment days year 1 after follow-up start	4697	75.59	73.40	77.79
Unemployment days year 2 after follow-up start	4697	50.82	48.75	52.88
Unemployment days year 3 after follow-up start	4697	39.26	37.30	41.21
Unemployment days year 4 after follow-up start	4697	31.76	29.96	33.55
**Reference population**				
Unemployment days the year of follow-up start	22013	18.41	17.80	19.03
Unemployment days year 1 after follow-up start	22013	16.86	16.25	17.48
Unemployment days year 2 after follow-up start	22013	13.64	13.08	14.20
Unemployment days year 3 after follow-up start	22013	12.16	11.62	12.71
Unemployment days year 4 after follow-up start	22013	10.20	9.70	10.70
**ASD**				
Unemployment days the year of follow-up start	814	40.11	35.11	45.11
Unemployment days year 1 after follow-up start	814	39.85	34.78	44.91
Unemployment days year 2 after follow-up start	814	30.62	26.12	35.13
Unemployment days year 3 after follow-up start	814	30.14	25.59	34.69
Unemployment days year 4 after follow-up start	814	29.28	24.48	34.09

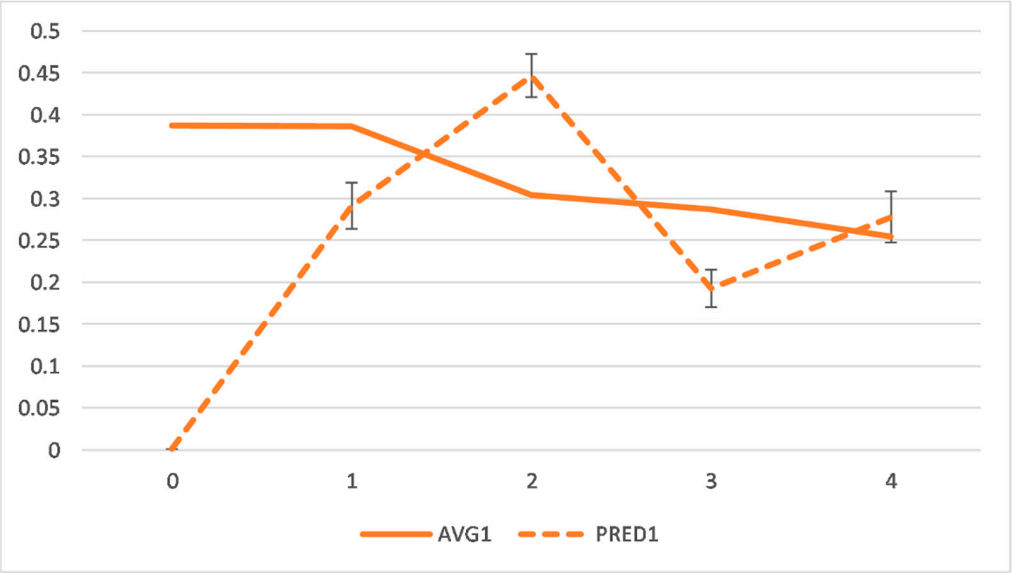

Figure S1. Unemployment trajectory during the diagnosis year (0) and a four-year follow-up among individuals with autism spectrum disorder (ASD, n=814). AVG=average (solid line), PRED= predicted for the identified trajectory group (dashed line with 95% confidence intervals). Cubic term was statistically significant for the shape of the trajectory.
